# 

**Published:** 1892-01-09

**Authors:** 


**Notices of Births, Deaths, and Marriages,** are inserted in this column at a charge of 2s. 6d. for an announcement not exceeding four lines.

### NOTICE TO CORRESPONDENTS.

All MS., Letters, Books for Review, and other matters intended for the Editor should be addressed THE EDITOR, THE LODGE, PORCHESTER SQUARE, LONDON, W.

The Editor cannot undertake to return rejected M.S., even when accompanied by stamped directed envelope.

**Notice to Advertisers and Subscribers.**

All Advertisements, Orders for Copies of THE HOSPITAL, and Business Communications should be addressed to The Publisher, *not to the Editor*, THE HOSPITAL, 140, Strand, W.C.

The Cost of Subscription, post free, is as follows:—Per week, 1½d.; per quarter, 1s. 8d.; per half-year, 3s. 3d.; per annum, 6s. 6d. Foreign:—Per annum, 8s. 8d.; payable, in all cases, in advance.

**"Burdett's Hospital Annual," 1891—1892.**

Third year of publication. 600 pp. Boards, gilt lettering. A most complete guide to British and Colonial Hospitals, Dispensaries. Nursing and Convalescent Institutions, and Asylums. Price 3s. 6d. Published by THE HOSPITAL (Limited), 140, Strand, W.C.

**NOTICE.—The Index for Vol. X. (ending September, 1891), is on sale at the Office of this Journal, price 2½d., post free.**

### GENERAL CHARITIES.

[This Column is devoted to an account of work of charitable institutions other than Medical Charities. The Editor will be glad to receive reports or news of work at 140, Strand. *Philanthropy* should be plainly written on the envelope.]

The Marchioness of Salisbury has become a patroness of the Home of Rest for Horses.

The Marquis of Dufferin and Ava has accepted the office of Vice-President of the Seamen's Hospital Society, in the room of the late Mr. W. H. Smith.

The Executive Committee of the **GORDON BOYS' HOME** are prepared to receive applications from gentlemen holding, or having held, a commission in the army or navy for the office of Commandant, which has become vacant.

The entertainments recently held on behalf of the **INVALID CHILDREN'S AID ASSOCIATION** proved most successful and satisfactory from every point of view. Over one hundred pounds were cleared from the proceeds of the ball, and the concert brought in sixty pounds.

Numerous hospitals and charitable institutions have benefited largely under the will of the late Mr. Robert Alger Newbon. The sum of £20,000 has been bequeathed to the Royal National Lifeboat Institution, and there are legacies of £1,000 each to several orphanages. In fact, hardly any institution of note seems to have been omitted under this generous will.

The Committee of the **SCHOOL FOR THE INDIGENT BLIND**, St. George's Fields, Southwark, appeal for funds on behalf of the Junior Branch School at Wandsworth. The blind people who receive the benefits of this society must be between the ages of seven and twenty; they are received for about six years, during which time they are taught a trade.

We regret to hear that funds are much needed to meet the current expenses of the training ships "Arethusa" and "Chichester," and the homes on shore under the management of the committee of the **NATIONAL REFUGES** for homeless boys and destitute children. Contributions sent to the Secretary, 164, Shaftesbury Avenue, W.C., will be thankfully received.

Among deserving institutions which will be grateful for assistance at this time of the year are the Royal Naval Benevolent Society, the object of which should appeal strongly to all officers of the Navy who have the means to afford assistance; the Shipwrecked Mariners' Society, the Newport Market Refuge, which has accommodation for one hundred destitute boys, but at present, owing to want of funds, can only support seventy-five; and the Mission to Deep Sea Fishermen, whose work is well-known, and whose benefits are sorely needed at this period of the year by a large class of hard workers.

It was asserted, not without some show of reason, at the second annual meeting the East London branch of the **SOCIETY FOR THE PREVENTION OF CRUELTY TO CHILDREN** that the existence of cruelty is frequently traceable to the fact that people have lost hold of the highest ideas of marriage. Many, as shown by the records of the Society, have unmistakably lost hold of all ideas of humanity, but it is not unreasonable to hope that the efforts of the Society, supported by a healthy public opinion, will in the long run root out all the miseries now entailed upon so many poor little sufferers. We think that a move has been made in the right direction by electing working men on the committee. Unfortunately this Society, like most others, is in need of funds; as its work appeals to all classes of people, it is to be hoped its needs will be duly recognised.

Christ Church parish, which is the district going north of Commercial Road, is probably the poorest in London, and it does not seem to have been discovered by those who shower their gifts of money and clothing on parts of East London where the need is infinitely less great. In this dreary district the rich are those who have been lucky enough to find a job; whilst their less fortunate neighbours are perpetually on the verge of starvation. The clergy are making a brave fight. One has only to walk through the streets with Mr. Littlewood, the curate, and see the way the children rush up to him and appreciate his efforts; but they cannot do single-handed what should be the work of willing helpers. Will those who can spare money, or blankets, or old clothes, or anything, or give any personal help, write to the Rev. A. B. Littlewood, 364, Commercial Road, E.?

The reports, which were presented at the recent distribution of prizes to the successful pupils of the **LONDON SOCIETY FOR TEACHING THE BLIND**, show that good work is being done by the Institution, where, in addition to being taught arithmetic, reading, and geography, the pupils are instructed in various industrial occupations. How necessary such work as is carried on by institutions of this class is is only too clearly proved by a remarkable passage in the report presented by the Hon. Chaplain to the effect that "in some cases the children who are admitted into the Institution have been left entirely uninstructed in religion, and have never even heard of Christ." At present there are nearly 60 pupils in the Institution, but much more can be done if the public will come forward with funds, and the Committee are anxious for enough money to support twenty-one more cots and to pay for the necessary bed clothing. Donations can be sent to Captain Webber, R.N., Secretary, at the Institution, Upper Avenue Road, St. John's Wood.

### SCRAPS AND GLEANINGS.

THE Queen has been graciously pleased to send a donation of £50 to the Bishop of London's Fund.

A FANCY DRESS BALL is to be held at Abergavenny on January 15th in in aid of the Cottage Hospital.

THE grand bazaar at Moscow will add some ten thousand pounds sterling to the Famine Relief Fund.

THE Earl of Dartmouth has accepted the office of President of the Royal Ear Hospital, in succession to the late Earl.

A CHARITABLE lady named Wallich, who died recently at Nice, has bequeathed £12,000 for the establishment of a hospital at Odessa.

THERE is a story going that a man in Vienna knocked six nails into his head in order to go to a hospital and have them taken out again.

THE Royal Free Hospital, Gray's Inn Road, has received a Christmas box of £146, the proceeds of a dance held in the Holborn Town Hall.

A GERMAN doctor reports that he has discovered the means by which the bacillus of influenza propagates. Prevention or cure would be more acceptable.

THE dissemination of influenza has, it is said, been distinctly traced to the circulation of Christmas cards. Several visitors from London at Hastings, have been attacked through this medium.

A NEW danger has arisen, if Dr. Granes, of Rhode Island, is to act as precedent to others. He obtained immense influence over his patient, induced her to consiitute him her legatee, and then deliberately poisoned her.

THROUGH the munificence of Lady Howard de Walden a children's ward, containing 14 beds, was opened by Sir W. Houldsworth on the 21st ult., at the Kilmarnock Infirmary. The plans were prepared by Mr. W. Railton.

THE promoters of the new Children's Hospital and Convalescent Home at Rhyl have received a communication from the Duke of Westminster promising £1,000 towards purchasing a site, and £2,000 towards the building fund.

A MEETING of the Secretaries and Treasurers of the Cork hospitals was held, when they presented their reports on the expenditure of their institutions, that an adequate share of the Hospital Saturday collection might be allotted to each.

THE Woodhall Spa Hospital is making good progress. The pretty little watering place is gaining a reputation for the curative qualities of its waters, and doubtless the number of paying patients at the hospital will continue to increase.

THE prevalence of influenza is more apparent day by day. At the Fulham Infirmary, help from outside, has been obtained, as the Superintendent and his assistants were working twelve hours a-day continuously. Abroad we hear of a like visitation of the epidemic.

HER Royal Highness Princess Beatrice has, with the assistance of the President and Vice-Presidents of the Berks and Bucks Needlework Guild, been able to send this winter nearly 2,000 useful garments to various districts in those counties for distribution among the deserving poor.

CHRISTMAS has been a busy time at the hospitals this year. St. Mary's received no less than twenty accident cases on Christmas Day, due to the slippery pavements. Since Christmas Eve ten broken legs have fallen to the lot of St. Bartholomew's, whilst Guy's reports a large number of fractures of the fore-arm.

THE Ayr County Hospital has passed through a year of activity and usefulness. The number of patients since 1883 have increased 50 per cent., and an outbreak of typhus during the year was most successfully grappled with. The hospital has lost a valued friend in Doctor Dobie, one of the visiting physicians.

As a local paper remarks Dumfermline hospital prospects gets "No for'ader." Indeed a dead block is threatened, as a proposal is being considered which provides for the utilisation of farm buildings for a temporary hospital at a cost of £300. We hope the canny Scot will show a little more wisdom and enterprise.

THE Cork Hospital Saturday Fund have distributed their late collection as follows:—North Infirmary £110; South Infirmary, £110; Mercy Hospital, £100; Women's and Children's Hospital, £60; Fever Hospital, £60; Eye, Ear, and Throat Hospital, £50; St. Vincent's Hospital, £40; Lying-in Hospital, £35; and the Cork Maternity £35.

AT a meeting of the Stratford and Avon Rural Sanitary Authorities, the proposal of joining with the Urban Sanitary Board for a joint infectious hospital was discussed. It was proposed in contradistinction to this scheme, to obtain several cottages to be held in readiness for purposes of isolation. No decision was, however, arrived at.

THE Mayor of Salford is much disappointed at the result of a special meeting on behalf of the Royal Infirmary. Only £805 was collected towards defraying the debt of £5,000. This does not certainly seem a very adequate sum for a neighbourhood reported to be so rich, and in consideration that Northampton has just presented its hospital with £1,170 as a voluntary gift.

THE usual decoration of the wards at the Great Northern Central Hospital, Holloway Road, N., was carried out this season, and the extra dinner provided for the patients and staff on Christmas Day. On January 5th the Ladies' Association gave an entertainment consisting of music and the exhibition of a magic lantern for the inmates, when a small present was given to each.

OWING to the exertions of Mr. E. C. Fenoulhit, a cottage hospital for Herne Bay was opened on the 1st ult. Cottages were bought at a cost of £400, raised by public subscription, which have been adapted without structural alterations for the purposes of the hospital. There will be four beds; patients will pay from 2s. 6d. to 10s. per week; and they will be received from a radius of about three miles.

THE St. John's Hospital Convalescent Home, Finchley Road, one of the only institutions of the kind, for patients suffering from diseases of the skin, has amply justified its establishment by the large numbers applying for admission. Although in the first instance it was started for patients of the St. John's Hospital, cases from other hospitals are now admitted. The house has been lent free of charge for one year by a member of the Board of Management.

### THE STUDENT OF MEDICINE.

[All communications for this Supplement should be addressed to THE EDITOR of THE HOSPITAL, at 140, Strand, with the words, "Students' Column," written in the left hand top corner of the envelope.]

### APPOINTMENTS.

J. M. Campbell, M.B., C.M. Glasg., has been appointed Medical Officer to the Workhouse at Caersws, Montgomeryshire. T. H. A. Chaplin, M.B. Cantab., M.R.C.P., has been appointed Pathologist to the City of London Hospital for Diseases of the Chest, Victoria Park. C. Clapham, M.D. Brux., M.R.C.P. Edin., L.R.C.P. Lond., M.R.C.S., has been appointed Honorary Physician to the Sheffield Public Hospital and Dispensary. J. E. Gabb, L.R.C.P., L.M. Edin., M.R.C.S., has been appointed Medical Officer of the Shrewsbury Dispensary. P. H. Gillies, M.B., C.M. Edin., has been appointed Junior Assistant Medical Officer to the Cumberland and Westmoreland Asylum. P. R. T. Harris, M.R.C.S., L.D.S., has been appointed Dental Surgeon to the Great Northern Central Hospital. S. G. M'Allum, M.B., has been appointed Junior Assistant Medical Officer to the County Asylum, Chester. G. R. Murray, B.A., M.B. Cantab., M.R.C.P. Lond., has been appointed Pathologist to the Hospital for Sick Children, Newcastle-on-Tyne. S. Roberts, M.A., M.B., B.C. Cantab., M.R.C.S., has been appointed Honorary Consulting Physician to the Sheffield Public Hospital and Dispensary. H. M. Speechly, M.R.C.S., L.R.C.P. Lond., has been reappointed Receiving-room Officer at the London Hospital. W. C. Swayne, M.B. Lond., M.R.C.S., L.R.C.P. Lond., has been appointed Honorary Obstetric Physician to the Royal Infirmary, Bristol. E. C. Williams, M.B. Cantab., has been appointed House Physician to the City of London Hospital for Diseases of the Chest, Victoria Park. J. W. Wyncoll, M.B., C.M. Edin., has been appointed Surgeon at the Dudley Dispensary. A. A. Bowlby, F.R.C.S., has been appointed an Assistant Surgeon to St. Bartholomew's Hospital. T. B. P. Davies, M.B., B.S. Lond., has been appointed Assistant House Surgeon to Guy's Hospital. Andrew Fullerton, M.B., B.Ch., B.A.O., has been reappointed Senior House Surgeon to the Miller Hospital and Royal Kent Dispensary. H. C. Garth, M.B., C.M., has been appointed Junior House Surgeon to the Miller Hospital and Royal Kent Dispensary, vice C. Evans, resigned. William F. Haslam, F.R.C.S., has been appointed Surgeon to the General Hospital, Birmingham. H. Hodgson, M.B., B.S. Lond., has been appointed House Surgeon to Guy's Hospital. H. Oswin Johnson, M.R.C.S., L.R.C.P., has been appointed House Surgeon to the Gloucester General Infirmary. G. L. Laycock, M.B., C.M. Edin., has been appointed Honorary Medical Officer to the Melbourne Children's Hospital, Victoria, Australia. D. S. Long, M.B., B.C. Cantab., has been appointed House Physician to Guy's Hospital. Allan E. Mahood, M.B., M.Ch., M.R.C.S., has been appointed House Surgeon to the Queen's Hospital, Birmingham. Archd. S. Percival, M.A., M.B., B.C. Camb., M.R.C.S. has been appointed Honorary Ophthalmic Surgeon to the Hospital for Sick Children, Newcastle-on-Tyne. W. G. Rogers, M.B. B.S. Lond., has been appointed House Surgeon to Guy's Hospital. R. H. Russell, L.R.C.P. Lond., F.R.C.S., has been appointed Honorary Medical Officer to the Melbourne Children's Hospital, Victoria, Australia. D. W. Samways, M.B., B.C. Cantab., has been appointed House Physician to Guy's Hospital. A. Thomas, M.B., B.S. Lond., has been appointed Assistant House Physician to Guy's Hospital. Robert Stanley Thomas, M.A., M.B., B.C. Cantab., has been appointed House Surgeon to the South Devon and East Cornwall Hospital, Plymouth. J. E. Thompson, M.B. Lond., C.M., M.R.C.S., has been appointed House Surgeon to the Wolverhampton Eye Infirmary. F. S. Wood, M.R.C.S., L.R.C.P., has been appointed Assistant House Surgeon to Guy's Hospital.

### VACANCIES.

### Resident Medical Officers.

City of London Hospital for Diseases of the Chest, Victoria Park.—Resident Medical Officer—Salary £100 per annum with board, &c.

Hospital for Sick Children, Great Ormond Street, London, W.C.—House Surgeon for one year—Salary £50 with board, &c.

Borough Hospital, Birkenhead, Junior House Surgeon—Salary £60, with board and lodging.

City of London Hospital for Diseases of the Chest, Victoria Park, E., Resident Medical Officer—Salary £100, with board, &c. (Apply to the Secretary, at the office, 24, Finsbury Circus, E.C.)

Holloway Sanatorium Hospital for the Insane, Virginia Water, Second Assistant Medical Officer—Salary £200, with furnished rooms and lodging.

Hospital for Sick Children, Great Ormond Street, London, W.C., House Surgeon for one year—Salary £50, with board and residence.

Royal Albert Hospital, Devonport, Assistant House Surgeon for six months—Board, lodging, and washing.

### The Hospital Advertiser.

MEDICAL APPOINTMENT.

THE HOSPITAL FOR SICK CHILDREN, GREAT ORMOND STREET, London, W.C.

